# Estimation of Percentage of Patients With Fibroblast Growth Factor Receptor Alterations Eligible for Off-label Use of Erdafitinib

**DOI:** 10.1001/jamanetworkopen.2019.16091

**Published:** 2019-11-22

**Authors:** Lelia Maria de Almeida Carvalho, Sandra de Oliveira Sapori Avelar, Alyson Haslam, Jennifer Gill, Vinay Prasad

**Affiliations:** 1 Hospital das Clínicas da Universidade Federal de Minas Gerais, Belo Horizonte, Minas Gerais, Brazil; 2 Knight Cancer Institute, Division of Hematology Oncology, Oregon Health and Science University, Portland; 3 Department of Public Health and Preventive Medicine, Oregon Health and Science University, Portland; 4 Center for Health Care Ethics, Oregon Health and Science University, Portland

## Abstract

**Importance:**

When a novel drug is granted accelerated approval, both its on-label and off-label uses must be taken into account.

**Objectives:**

To estimate the potential upper bound of off-label use of erdafitinib to treat advanced cancer with fibroblast growth factor receptor gene (*FGFR*) alterations, compare it to the upper bound of on-label use in urothelial cancer, and to review studies that may support off-label use.

**Design, Setting, and Participants:**

This cross-sectional study used frequency data on *FGFR* alterations by cancer type and the estimated number of deaths from all cancers for 2019 in the United States. Mortality statistics were used as surrogates for patients with advanced cancer. Analysis was conducted in May 2019.

**Exposure:**

Percentage of patients with an *FGFR2* or *FGFR3* alteration.

**Main Outcomes and Measures:**

Estimated number of patients with advanced cancer expressing an *FGFR2* or *FGFR3* alteration eligible for off-label use of erdafitinib by cancer type; number of studies investigating *FGFR*-targeting drugs for patients with cancer; and number of ongoing clinical trials on erdafitinib by cancer type.

**Results:**

A total of 15 cancer types had reported *FGFR* alterations. Of 455 440 estimated patients who died of cancer in 2019, 17 019 (3.7%) were estimated to have *FGFR2* or *FGFR3* alterations. Of these patients, 12 955 (76.1%) could be eligible for off-label treatment with erdafitinib. A total of 29 completed studies evaluated *FGFR*-targeting drugs in 11 cancer types, and 10 ongoing studies are studying erdafitinib for different oncological indications.

**Conclusions and Relevance:**

This study indicates that the potential for off-label use of *FGFR* inhibitors such as erdafitinib spans a number of cancer types and a large patient population. Systematic trials exploring off-label uses may be desirable for drugs that target clear, identifiable molecular alterations because this may be more efficient than off-label use in identifying clinical scenarios where the agent has activity.

## Key Points

**Question:**

What is the potential upper bound of off-label use of erdafitinib in cancers with fibroblast growth factor receptor (*FGFR*) alterations?

**Findings:**

In this cross-sectional study, an estimated 76.1% of patients with advanced cancer expressing *FGFR2* or *FGFR3* alterations could be eligible for off-label treatment with erdafitinib. A total of 29 completed studies evaluating *FGFR*-targeting drugs in 11 cancer types and 10 ongoing studies on erdafitinib for different oncological indications were identified.

**Meaning:**

This study indicates that the potential for off-label use of *FGFR* inhibitors, such as erdafitinib, spans a number of cancer types and a large patient population.

## Teaser

This cross-sectional study estimates the potential upper bound of off-label use of erdafitinib to treat advanced cancer with fibroblast growth factor receptor (*FGFR*) gene alterations, compares it to the upper bound of on-label use in urothelial cancer, and reviews studies that may support off-label use.

## Introduction

Erdafitinib was recently granted accelerated approval by the US Food and Drug Administration (FDA) for the treatment of patients with locally advanced or metastatic urothelial cancer with fibroblast growth factor receptor 2 (*FGFR2*) or *FGFR3* gene mutations or fusions.^1^ Erdafitinib targets *FGFR2* and *FGFR3*, receptors commonly expressed in metastatic urothelial cancer, and belongs to the more general class of tyrosine kinase inhibitors.^2^ It was the first *FGFR*-targeting drug approved by the FDA. The approval of erdafitinib was based on overall response rate (ORR) in 87 patients with *FGFR2* and *FGFR3* alterations from a single-group, phase 2, multicenter study.^2,3^ Among responders, median (interquartile range) duration of response was found to be 5.4 (4.2-6.9) months. The response rate varied considerably by alteration, with an ORR of 40.6% (26 of 64) for *FGFR3* point mutations, 11.1% (2 of 18) for *FGFR3* fusions, and 0% (0 of 6) for *FGFR2* fusions.^3^

Urothelial cancer is not the only cancer type that harbors *FGFR* alterations, which may be found in breast cancer, non–small cell lung cancer, colorectal cancer, and endometrial cancer, among others.^4^ The availability of a drug targeting *FGFR2* and *FGFR3* alterations for 1 tumor type (ie, urothelial cancer) may encourage the off-label use in other types of cancers with these alterations. Patients with tumor types other than urothelial cancer already have access to erdafitinib through the expanded access program,^5^ and enthusiasm for precision therapies is high. Other studies have reported broad-based sequencing and off-label use of tyrosine kinase inhibitor paid for by insurers.^6^ Finally, empirical analyses show that molecularly targeted drugs are often recommended by expert panels for tumor types different from those that received approval.^7^

This study aimed to estimate the potential upper bound of off-label use of erdafitinib to treat other types of advanced cancer with *FGFR* alterations, determine an estimated ratio of off-label use to on-label use, and review studies that may support the benefit of off-label use.

## Methods

### Overview

In this cross-sectional study, we sought to estimate what percentage of *FGFR2* and *FGFR3* mutations and fusions were in approved vs unapproved tumor types for the drug erdafitinib. We also sought to document available, corroborative, or circumstantial evidence supporting the benefit of using erdafitinib to treat off-label tumor types.

Per Oregon Health and Science University human research protection program policy,^8^ this study did not require institutional review board approval as it did not involve personally identifiable data and all data are publicly available. This report followed the Strengthening the Reporting of Observational Studies in Epidemiology (STROBE) reporting guideline.

### Estimates

We extracted cancer-specific *FGFR* aberration frequency data by histology from Helsten et al.^4^ We obtained the estimated number of deaths from all cancers from the *American Cancer Society: Cancer Facts and Figures 2019*.^9^ Cancer types were included in the analysis if found in data sets from Helsten et al^4^ and *Cancer Facts and Figures 2019*.^9^ We used mortality statistics as a surrogate for incident presentation of advanced or metastatic cancer. To determine the upper-bound number of patients eligible for off-label use of erdafitinib by cancer type, the number of cancer deaths was multiplied by the percentage of patients who had an *FGFR2* or *FGFR3* mutation or fusion for each cancer type. This process was replicated for patients with any *FGFR* alteration. By determining the number of cancer patients in each cancer type with any *FGFR* alteration, we sought to offer a second, broader estimation of potential eligibility for off-label treatment with erdafitinib.

Off-label use was defined as any use of erdafitinib for cancer types other than urothelial cancer. We determined off-label eligibility specifically for *FGFR2* and *FGFR3* alterations because erdafitinib was approved for these alterations in urothelial cancer. Our methods were similar to prior analyses of the estimated, upper-bound effect of genome-guided therapies^10^ and immunotherapy checkpoint inhibitors^11^ in cancer medicine.

### Studies Targeting *FGFR* Alterations in Other Cancer Types

To review studies that may be used to support off-label use of erdafitinib, we searched PubMed for studies investigating therapies targeting *FGFR* alterations in cancer types other than urothelial cancer. To search PubMed, we used the article type filters of *case reports*, *clinical study*, and *clinical trial* and searched the phrase *FGFR* with 1 of the following cancer types: carcinoma of unknown primary site, non–small cell lung cancer, pancreatic exocrine cancer, breast cancer, endometrial cancer, colorectal cancer, glioma, head and neck squamous cell cancer, gastric or gastroesophageal junction cancer, ovarian cancer, renal cell cancer, cholangiocarcinoma, all sarcomas, and melanoma. All studies investigating the use of an *FGFR*-targeting drug to treat cancer patients were included. Articles investigating mouse models or in vivo studies were excluded as well as articles assessing *FGFR* alterations in patients without investigating a *FGFR*-targeting treatment. Data collected included the title of study, study type (case report, case series, or phase 1, 2, or 3 trial), randomization, primary outcome, and number of participants.

For ongoing trials of erdafitinib, a search was made on ClinicalTrials.gov using the term *erdafitinib*. Results were filtered by excluding trials that were suspended, terminated, completed, or withdrawn. We also excluded studies in healthy patients and registered studies that did not have an intervention (eg, estimating eligibility). Searches of PubMed and ClinicalTrials.gov were made on June 6, 2019.

### Statistical Analysis

We generated an estimate of the percentage of patients in the United States with cancer and *FGFR* alterations eligible for on-label and off-label treatment with erdafitinib by cancer type. All statistical analyses were conducted in Excel 2016 (Microsoft). The reviews of studies on targeting *FGFR* alterations and ongoing studies of erdafitinib were purely descriptive. Because all statistics are descriptive, no prespecified level of statistical significance was set. The study was conducted in May 2019.

## Results

### Estimation of Patients Eligible for Off-label Treatment With Erdafitinib, Based on *FGFR2* and *FGFR3* Alterations

An estimated 455 440 individuals with cancer died in 2019. Of those, 17 019 (3.7%) were estimated to have either an *FGFR2* or an *FGFR3* alteration. A total of 15 cancer types, including urothelial cancer, had reported *FGFR* alterations (Table 1). Among the cancer types, urothelial cancer, carcinoma of unknown primary, non–small cell lung cancer, pancreatic exocrine cancer, and breast cancer had the highest number of patients with *FGFR2* or *FGFR3* alterations (urothelial cancer, 4064 of 17 670 patients [23.0%]; carcinoma of unknown primary sites, 2708 of 45 140 [6.0%]; non–small cell lung cancer 2140 of 142 670 [1.9%]; pancreatic exocrine cancer, 1601 of 45 750 [3.5%]; breast cancer, 1310 of 42 260 [3.1%]). We estimated that, of 17 019 patients with advanced cancer expressing *FGFR2* or *FGFR3* alterations, 12 955 (76.1%) could be eligible for off-label treatment with erdafitinib (Figure).

**Table 1.** Frequencies of *FGFR* Mutations and Fusions in the United States

Cancer Type	Estimated No.		
	Cancer Deaths, 2019 (N = 455 440)	Patients With *FGFR* Alteration (n = 35 536)^a^	Patients With *FGFR2* or *FGFR3* Alterations (n = 17 019)^a^
Urothelial cancer	17 670	5601	4064
Carcinoma of unknown primary site	45 140	3701	2708
Non–small cell lung cancer	142 670	7418	2140
Pancreatic exocrine cancer	45 750	2150	1601
Breast cancer	42 260	7353	1310
Endometrial cancer	12 160	1374	1216
Colorectal cancer	52 300	2301	889
Glioma	17 760	1350	746
Head and neck squamous cell cancer	21 720	999	586
Gastric or GE junction cancer	11 140	746	546
Ovarian cancer	13 980	1202	476
Renal cell cancer	14 770	679	340
Cholangiocarcinoma	3960	277	242
Sarcoma, all	6930	277	104
Melanoma	7230	108	51

Abbreviations: *FGFR*, fibroblast growth factor receptor gene; GE, gastroesophageal.

^a^ Based on percentages of *FGFR* alterations reported in Helsten et al.^4^

**Figure.**
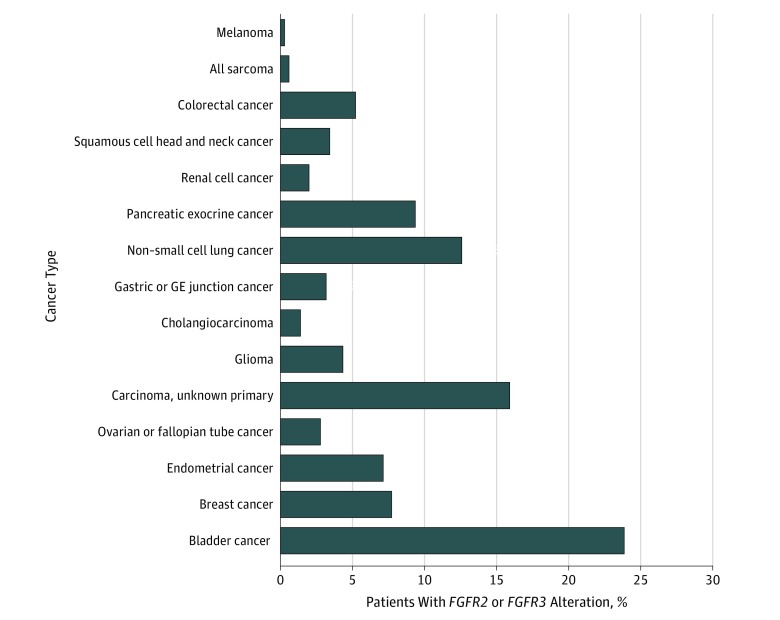
Estimated Number of Individuals With Fibroblast Growth Factor Receptor 2 (*FGFR2*) and *FGFR3* Alterations Who Could Be Eligible for Off-label Use of Erdafitinib GE indicates gastroesophageal.

### Estimation of Patients Eligible for Off-label Treatment With Erdafitinib Based on Any *FGFR* Alteration

We also estimated the percentage of patients eligible for treatment with erdafitinib among a pool of patients with any *FGFR* alteration (ie, not necessarily *FGFR2* or *FGFR3*) (eFigure in the Supplement). Among 455 440 estimated patients, 35 536 (7.8%) had any FGFR alteration, and of these patients, 5601 (15.8%) had urothelial cancer. Therefore, if treatment were targeted at any *FGFR* alteration, an estimated 84.2% of the potential use of *FGFR* inhibitors, such as erdafitinib, would be off-label.

### Studies Evaluating Erdafitinib and Other *FGFR*-Targeting Drugs in Various Cancer Types

We found 29 completed studies evaluating *FGFR*-targeting drugs in 11 of 14 cancer types analyzed in the off-label estimations (Table 2).^12,13,14,15,16,17,18,19,20,21,22,23,24,25,26,27,28,29,30,31,32,33,34,35,36,37,38^ There were 8 (27.6%) case reports, 1 (3.4%) case series, 9 (31.0%) phase 1 studies, 2 (6.9%) phase 1/2 studies, and 9 (31.0%) phase 2 studies. While 2 phase 2 trials (22.2%) were randomized, the rest were noncomparator trials. All phase 1 studies had primary outcomes of either safety or dosage. Phase 2 trials evaluated ORR (5 studies [55.5%]), progression-free survival (4 studies [44.4%]), or event-free survival (1 study [11.1%]) as their primary outcome.

**Table 2.** Studies Evaluating *FGFR*-Targeting Drugs in Other Cancer Types

Cancer Type	Study Title	Study Type	Patients, No.	Primary Outcome
Breast cancer	Pazopanib Sensitivity in a Patient With Breast Cancer and *FGFR1* Amplification^12^	Case report	1	NA
Breast cancer	Phase II, Randomized, Placebo-Controlled Study of Dovitinib in Combination With Fulvestrant in Postmenopausal Patients With HR^+^, HER2^-^ Breast Cancer That Had Progressed During or After Prior Endocrine Therapy^13^	Phase 2, randomized	97	PFS
Breast cancer	Phase I/IIa Study Evaluating the Safety, Efficacy, Pharmacokinetics, and Pharmacodynamics of Lucitanib in Advanced Solid Tumors^14^	Phase 1/2, nonrandomized	76	Dosage
Breast cancer	Targeting *FGFR* With Dovitinib (TKI258): Preclinical and Clinical Data in Breast Cancer^15^	Phase 2 nonrandomized	81	ORR
Non–small cell lung cancer	A Phase 1 Study of LY2874455, an Oral Selective Pan-*FGFR* Inhibitor, in Patients With Advanced Cancer^16^	Phase 1, nonrandomized	36	Dosage
Non–small cell lung cancer	Evaluation of BGJ398, a Fibroblast Growth Factor Receptor 1-3 Kinase Inhibitor, in Patients With Advanced Solid Tumors Harboring Genetic Alterations in Fibroblast Growth Factor Receptors: Results of a Global Phase I, Dose-Escalation and Dose-Expansion Study^17^	Phase 1, nonrandomized	132	Dosage
Non–small cell lung cancer	Efficacy and Safety of Dovitinib in Pretreated Patients With Advanced Squamous Non–Small Cell Lung Cancer With *FGFR1* Amplification: A Single-Arm, Phase 2 Study.^18^	Phase 2, nonrandomized	26	ORR
Endometrial cancer	Comprehensive Genomic Profiling Aids in Treatment of a Metastatic Endometrial Cancer^19^	Case report	1	NA
Endometrial cancer	Phase I Dose-Escalation Study of JNJ-42756493, an Oral Pan–Fibroblast Growth Factor Receptor Inhibitor, in Patients With Advanced Solid Tumors^20^	Phase 1, nonrandomized	65	Safety, dosage
Endometrial cancer	A Phase II Evaluation of Nintedanib (BIBF-1120) in the Treatment of Recurrent or Persistent Endometrial Cancer: An NRG Oncology/Gynecologic Oncology Group Study^21^	Phase 2, nonrandomized	32	EFS
Endometrial cancer	A Phase II Trial of Brivanib in Recurrent or Persistent Endometrial Cancer: An NRG Oncology/Gynecologic Oncology Group Study.^22^	Phase 2, nonrandomized	43	PFS, ORR
Colorectal cancer	A Phase I Dose–Escalation Study of Regorafenib (BAY 73–4506), an Inhibitor of Oncogenic, Angiogenic, and Stromal Kinases, in Patients With Advanced Solid Tumors^23^	Phase 1, nonrandomized	16	Dosage
Glioma	Phase I Trial of Dovitinib (TKI258) in Recurrent Glioblastoma^24^	Phase 1, nonrandomized	12	Safety
Glioma	Detection, Characterization, and Inhibition of *FGFR–TACC* Fusions in IDH Wild-Type Glioma^25^	Case report	2	NA
Glioma	Phase II Trial of Triple Tyrosine Kinase Receptor Inhibitor Nintedanib in Recurrent High-Grade Gliomas^26^	Phase 2, nonrandomized	22	PFS
Glioma	Phase II Open-Label Study of Nintedanib in Patients With Recurrent Glioblastoma Multiforme^27^	Phase 2, nonrandomized	25	ORR
Gastric or GE junction cancer	Gastric Perforation Related to Concurrent Use of Nintedanib and Ramucirumab^28^	Case report	1	NA
Gastric or GE junction cancer	A Randomized, Open-Label Study of the Efficacy and Safety of AZD4547 Monotherapy vs Paclitaxel for the Treatment of Advanced Gastric Adenocarcinoma With *FGFR2* Polysomy or Gene Amplification^29^	Phase 2, randomized	71	PFS
Gastric or GE junction cancer	A Phase 1 Study of LY2874455, an Oral Selective Pan-FGFR Inhibitor, in Patients With Advanced Cancer^16^	Phase 1, nonrandomized	36	Dosage
Gastric or GE junction cancer	Acquired Resistance to LY2874455 in *FGFR2*-Amplified Gastric Cancer Through an Emergence of Novel *FGFR2-ACSL5* Fusion^30^	Case report	1	NA
Ovarian cancer	Tumors With *AKT1E17K* Mutations Are Rational Targets for Single Agent or Combination Therapy With AKT Inhibitors^31^	Phase 1, nonrandomized	41	Safety
Renal cell cancer	Phase II Results of Dovitinib (TKI258) in Patients With Metastatic Renal Cell Cancer^32^	Phase 2, nonrandomized	67	ORR
Renal cell cancer	Phase I Study of Dovitinib (TKI258), an Oral *FGFR*, *VEGFR*, and *PDGFR* Inhibitor, in Advanced or Metastatic Renal Cell Carcinoma^33^	Phase 1, nonrandomized	20	Dosage
Renal cell cancer	A Phase I Dose-Escalation Study of Regorafenib (BAY 73-4506), an Inhibitor of Oncogenic, Angiogenic, and Stromal Kinases, in Patients With Advanced Solid Tumors^23^	Phase 1, nonrandomized	53	Dosage
Renal cell cancer	*FGFR *Inhibitor Induced Peripheral Neuropathy in Patients With Advanced RCC^34^	Case report	1	NA
Cholangiocarcinoma	Polyclonal Secondary *FGFR2* Mutations Drive Acquired Resistance to *FGFR* Inhibition in Patients With *FGFR2* Fusion-Positive Cholangiocarcinoma^35^	Case series	3	NA
Sarcoma, all	Clinical Benefit of Pazopanib in a Patient With Metastatic Chondrosarcoma: A Case Report and Review of the Literature^36^	Case report	1	NA
Melanoma	Calcinosis Cutis Dermatologic Toxicity Associated With Fibroblast Growth Factor Receptor Inhibitor for the Treatment of Wilms Tumor^37^	Case report	1	NA
Melanoma	Phase I/II and Pharmacodynamic Study of Dovitinib (TKI258), an Inhibitor of Fibroblast Growth Factor Receptors and *VEGF* Receptors, in Patients With Advanced Melanoma^38^	Phase 1/2, nonrandomized	47	Safety, ORR

Abbreviations: AKT, protein kinase B; HER, human epidermal growth factor; HR, hormone receptor; IDH, isocitrate dehydrogenase; EFS, event-free survival; *FGFR*, fibroblast growth factor receptor; GE, gastroesophageal; NA, not applicable; ORR, overall response rate; *PDGFR*, platelet-derived growth factor receptor; PFS, progression-free survival; RCC, renal cell cancer; *TACC*, transforming acidic coiled-coil; *VEGF*, vascular endothelial growth factor; *VEGFR*, vascular endothelial growth factor receptor.

There were 10 ongoing studies of erdafitinib being used for oncological indications registered on ClinicalTrials.gov, 2 (20.0%) of which included patients with urothelial cancer (eTable in the Supplement). There were 2 (20.0%) phase 1 studies, 2 (20.0%) phase 1/2 studies, 5 (50.0%) phase 2 studies, and 1 (10.0%) phase 3 study. One study (10.0%) was evaluating overall survival as its primary outcome; the rest were evaluating ORR (7 studies [70.0%]) or safety (3 studies [30.0%]). Two of the 10 trials (20.0%) were randomized.

## Discussion

From our estimates, the number of patients with tumors exhibiting *FGFR2* or *FGFR3* alterations who may be potentially eligible for off-label treatment with an *FGFR* inhibitor is 3 times that of those eligible for on-label treatment. If interpretation of off-label use is understood more broadly to cover patients with any *FGFR* alteration, potential eligibility for off-label use would grow to be 5 times greater than on-label use. Alterations in *FGFR* are being studied in a number of cancers where these alterations are prevalent, and ORRs similar to that of erdafitinib in urothelial cancer may further encourage off-label treatments in other cancer types. Off-label use of targeted therapies is widely practiced in oncology.^7,39^ Erdafitinib was provisionally approved based on a surrogate outcome in a nonrandomized trial, and proof of clinical benefit will only be known after a confirmatory randomized trial investigating overall survival is conducted. The trial which led to the approval of erdafitinib found that adverse reactions of grade 3 or higher were reported by 67% of participants, and 46% were considered by investigators to be related to treatment.^2^ The FDA determined that the interim surrogate outcomes and toxic effects of erdafitinib were appropriate for patients with advanced urothelial cancer who had an *FGFR2* or *FGFR3* mutation or fusion. However, by approving this drug for this indication, the FDA has inevitably permitted off-label use of erdafitinib in many cancer types and among a considerably larger patient population.

### Strengths and Limitations

There are strengths and limitations in our analysis. One strength is that we sought to capture the potential on-label and off-label use of a novel genome-targeted drug entering the US marketplace, and we estimated the ratio of potential on-label to off-label use. To our knowledge, this is the first article that seeks to do this at the time of product launch. Moreover, we compiled data that clinicians could use to justify off-label use (Table 2). We note that these studies are largely exploratory in nature and reliant on small uncontrolled studies, which are generally considered to be at the bottom of the hierarchy of medical evidence.^40^

There are also limitations. We determined cancer-specific *FGFR* aberration frequencies through an analysis that looked at next-generation sequencing from 1 company,^4^ which may not be wholly representative of population-level frequencies. We used cancer deaths to estimate cases of metastatic disease in each cancer type, which is an imperfect surrogate. Cancer deaths are not all because of metastatic disease, and as patients with metastatic disease live longer, the incidence of metastatic cancer cases outpaces the incidence of cancer deaths. Unfortunately, to our knowledge, there are currently no data on metastatic disease in the United States, as staging data are only recorded at diagnosis. Nevertheless, we found that cancer death data were the best available option to explore our study objective. A study using the Cancer Registry of Norway^41^ found that the majority of cancer deaths were because of metastatic disease, and the recorded numbers are likely underestimated because of underreporting of metachronous metastases. If data on metastatic status are collected in the US population in the future, we encourage researchers exploring this topic to use incidence of metastatic cancer instead.

We have almost certainly overestimated the number of cases of metastatic disease and, within that number, the number of patients who would be treated with an off-label drug. This assessment was not conducted to accurately approximate the number of patients who will be treated with erdafitinib but instead to determine the proportion of patients eligible for off-label use and to highlight the fact that the consequences of this accelerated approval reach far beyond the patient population it was intended to treat. Off-label drug use is practiced across almost all cancer types, most likely prescribed to metastatic cancer patients in a later line because of unapproved indication for specific tumors.^42^ Completed and ongoing trials on erdafitinib and other *FGFR*-targeting drugs in various cancers exemplify the interest around this type of targeted therapy, and the fact that erdafitinib is the first *FGFR*-targeting drug to be approved by the FDA^43^ makes it susceptible to off-label use for patients with *FGFR* alterations. Enthusiasm for precision oncology may fuel this extrapolation.^44,45,46,47^

## Conclusions

This study found that the potential for off-label use of *FGFR* inhibitors such as erdafitinib spans a number of cancer types and a large patient population. Off-label use may be 3-fold higher than on-label use, based on population cancer statistics and the frequency of molecular alterations. Clinicians interested in off-label use may easily find case reports or series that may provide exploratory or circumstantial support for those choices. Because it may be tempting and plausible to use *FGFR* inhibitors for nonapproved uses, our analysis suggests that the size of this market share may be several times larger than the approved indication. Systematic trials to explore off-label uses may be desirable for drugs that target clear, identifiable molecular alterations because this may be more efficient than off-label use in identifying clinical scenarios where this agent has activity.

## Notes

Supplement. **eFigure. **Estimated Number of Individuals With Any *FGFR* Alteration and Percentage of Eligibility That Is Attributed to Approved or Off-label Use of *FGFR* Inhibitors **eTable. **Ongoing Studies of Erdafitinib Registered Through ClinicalTrials.gov
