# Supplementary material for: Estimation of Percentage of Patients With Fibroblast Growth Factor Receptor Alterations Eligible for Off-label Use of Erdafitinib
Source: JAMA Netw Open. 2019 Nov 22;2(11):e1916091. doi: 10.1001/jamanetworkopen.2019.16091 (PMC6902826; doi:10.1001/jamanetworkopen.2019.16091)
Supplement: Supplement. — eFigure. Estimated Number of Individuals With Any FGFR Alteration and Percentage of Eligibility That Is Attributed to Approved or Off-label Use of FGFR Inhibitors eTable. Ongoing Studies of Erdafitinib Registered Through ClinicalTrials.gov [file jamanetwopen-2-e1916091-s001.pdf]

# Supplementary Online Content

de Almeida Carvalho LM, de Oliveira Sapori Averlar S, Haslam A, Gill J, Prasad V. Estimation of percentage of patients with fibroblast growth factor receptor alterations eligible for off-label use of erdafitinib. *JAMA Netw Open*. 2019;2(11):e1916091. doi:10.1001/jamanetworkopen.2019.16091

**eFigure.** Estimated Number of Individuals With Any *FGFR* Alteration and Percentage of Eligibility That Is Attributed to Approved or Off-label Use of *FGFR* Inhibitors
**eTable.** Ongoing Studies of Erdafitinib Registered Through ClinicalTrials.gov

This supplementary material has been provided by the authors to give readers additional information about their work.

© 2019 de Almeda Carvalho LM et al. *JAMA Network Open.*

**eFigure. Estimated Number of Individuals With Any *FGFR* Alteration and Percentage of Eligibility That Is Attributed to Approved or Off-label Use of *FGFR* Inhibitors**

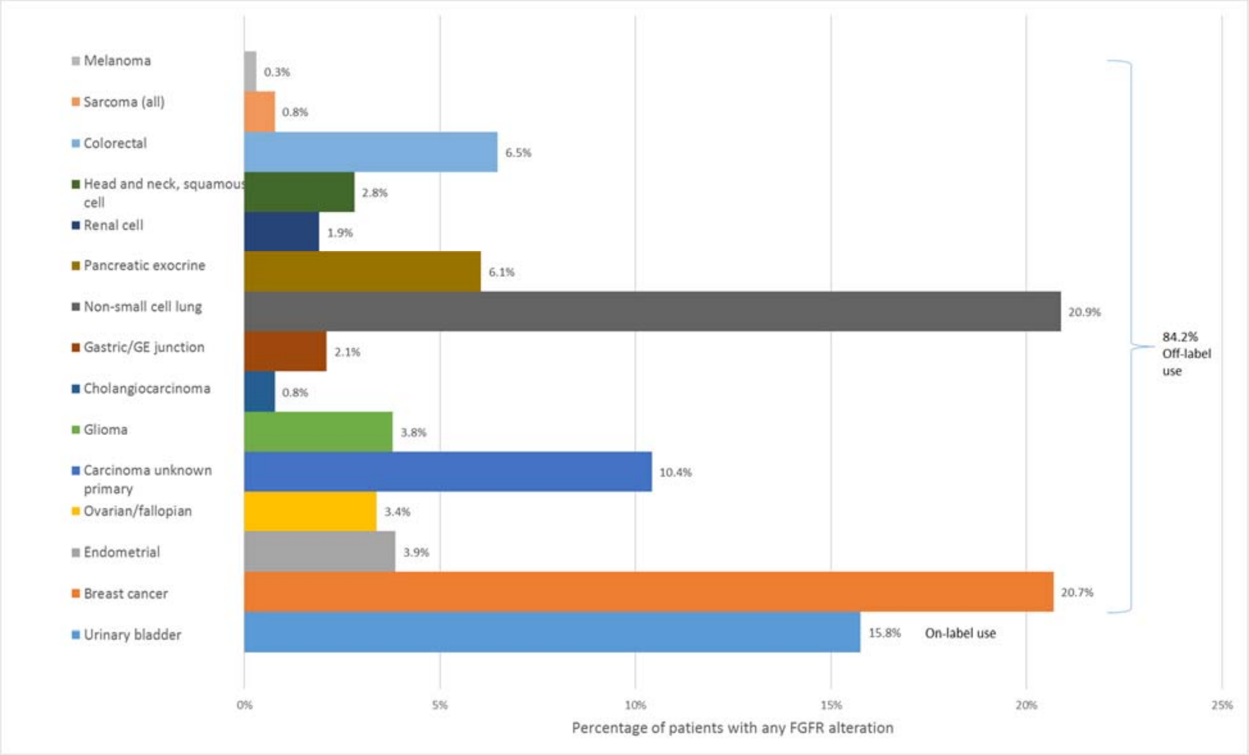


© 2019 de Almeda Carvalho LM et al. *JAMA Network Open*.

**eTable. Ongoing Studies of Erdafitinib Registered Through ClinicalTrials.gov**

| Cancer Type | Title of Case Study/trial | Type of study | Primary outcome | Start date-estimated completion date | # of Patients in trial | Clinical trials NCT# |
|---|---|---|---|---|---|---|
| Advanced solid cancers | A Study to Evaluate the Safety, Pharmacokinetics, Pharmacodynamics, and Immunogenicity of JNJ-63723283, an Anti-Programmed Cell Death (PD)-1 Monoclonal Antibody, as Monotherapy or in Combination With Erdafitinib in Japanese Participants With Advanced Solid Cancers | Phase I, non randomized | Safety | August 31, 2018 – July 8, 2021 | 30 | NCT03547037 |
| Urothelial | A Study to Evaluate Safety, Efficacy, Pharmacokinetics, and Pharmacodynamics of Erdafitinib Plus JNJ-63723283, an Anti-PD-1 Monoclonal Antibody, in Participants With Metastatic or Surgically Unresectable Urothelial Cancer With Selected FGFR Gene Alterations | Phase I/II, randomized | Safety and ORR | April 5, 2018 – June 17, 2021 | 102 | NCT03473743 |
| Advanced malignant solid neoplasm | Erdafitinib in Treating Patients With Relapsed or Refractory Advanced Solid Tumors, Non-Hodgkin Lymphoma, or Histiocytic Disorders With FGFR Mutations (A Pediatric MATCH Treatment Trial) | Phase II, non-randomized | ORR | Nov 6, 2017 – December 31, 2024 | 49 | NCT03210714 |
| Neoplasm | A Study to Evaluate the Clinical Efficacy of JNJ-42756493 (Erdafitinib), A Pan-Fibroblast Growth Factor Receptor (FGFR) Tyrosine Kinase Inhibitor, In Asian Participants With Advanced Non-Small-Cell Lung Cancer, Urothelial Cancer, Esophageal Cancer Or Cholangiocarcinoma | Phase II, non-randomized | ORR | July 8, 2016 – August 31, 2020 | 55 | NCT02699606 |
| Breast | Fulvestrant, Palbociclib and Erdafitinib in ER+/HER2-/FGFR-amplified Metastatic Breast Cancer | Phase I, non-randomized | Safety | August 10, 2017 – September 2022 | 32 | NCT03238196 |
| Urothelial | A Study of Erdafitinib Compared With Vinflunine or Docetaxel or Pembrolizumab in Participants | Phase III, | OS | March 23, 2018 – | 631 | NCT03390504 |

© 2019 de Almeda Carvalho LM et al. *JAMA Network Open.*

| | | | | | | |
|---|---|---|---|---|---|---|
| | With Advanced Urothelial Cancer and Selected Fibroblast Growth Factor Receptor (FGFR) Gene Aberrations | randomized | | November 5, 2021 | | |
| Lung | FGFR Inhibitor in FGFR Dysregulated Cancer | Phase II, non-randomized | ORR | March 15, 2019 – March 15, 2022 | 50 | NCT03827850 |
| Multiple myeloma | Myeloma-Developing Regimens Using Genomics (MyDRUG) | Phase I/II, non-randomized | ORR | April 1, 2019 – February 10, 2024 | 228 | NCT03732703 |
| Advanced malignant neoplasm | Targeted Therapy Directed by Genetic Testing in Treating Patients With Advanced Refractory Solid Tumors, Lymphomas, or Multiple Myeloma (The MATCH Screening Trial) | Phase II, non-randomized | ORR | August 12, 2015 – June 30, 2022 | 6452 | NCT02465060 |
| Advanced malignant neoplasm | Targeted Therapy Directed by Genetic Testing in Treating Pediatric Patients With Relapsed or Refractory Advanced Solid Tumors, Non-Hodgkin Lymphomas, or Histiocytic Disorders (The Pediatric MATCH Screening Trial) | Phase II, non-randomized | ORR | July 24, 2017 – September 30, 2027 | 1000 | NCT03155620 |

© 2019 de Almeda Carvalho LM et al. *JAMA Network Open*.
